# Observations on a Peculiar Species of Convulsions in Children

**Published:** 1825-01

**Authors:** John North

**Affiliations:** Surgeon.


### ORIGINAL COMMUNICATIONS,

### SELECT OBSERVATIONS, &c.

ART. I.—*Observations on a peculiar Species of Convulsions in Children.* By JOHN NORTH, Surgeon.*

I AM perfectly aware that the opinions I have ventured to state in the following observations, are at variance with those which are entertained upon the same subject by many highly respectable practitioners. I do not think it necessary, however, to preface any laboured apology for the free expression of opinions, which the repeated observation of facts has induced me to believe are well founded. I am conscious of no impropriety in respectfully questioning the doctrines of any man, when they appear to be erroneous in principle and dangerous in practice. It is not my intention to enter into a general description of the convulsive affections of children, although, perhaps, a concentrated and practical view of the subject is much to be desired. I shall confine myself to one particular species, of which I have seen several examples.

The symptoms which I am about to detail, very nearly resemble those which have been described by the late Dr. JOHN CLARKE.† The premonitory symptoms occur at an uncertain age,—generally, I believe, between the third and seventh month. At first, they may not be sufficiently striking to attract the particular attention of the friends, although the practitioner, who has had opportunities of watching the progress of similar cases, might, with much confidence, predict the series of symptoms which is yet to be developed. Each time the child wakes from its sleep, the breathing is for some moments unusually accelerated, and is accompanied by such a kind of noise as would be caused by an increased secretion of the mucus of the aerial passages. If the little patient has previously enjoyed a good state of health, the characteristic rotundity of features observable in the infantile state, will quickly undergo a remarkable change. The countenance soon becomes anxious; the sides of the nose are drawn in; the face is pallid and emaciated. When put to the breast, the child sucks greedily for a moment, but suddenly ceases to do so, and frequently throws back the head, which

* The subject of Mr. NORTH's paper has lately occupied the attention of a Medical Society in this metropolis. During the discussion which took place, the doctrine he deprecates was very strenuously supported by some members, whilst by others it was as warmly opposed.—EDITORS.

† *Commentaries on Diseases of Children,* chap. iv.

remains rigidly extended for some time. Whatever may have been the previous condition of the bowels, they now become constipated.

This state may continue for a very uncertain time, without any remarkable alteration: the following symptoms, however, are gradually added to those above enumerated; they occur irregularly. A convulsive affection of the hand is usually the next morbid sign which excites attention. The child's thumb will be found constantly and firmly pressed upon the palm of the hand. The wrist and ancle joints are bent rigidly inwards. The head is now almost constantly thrown backwards, keeping the anterior muscles of the neck upon the stretch. The inconvenience the child suffers when he wakes, is no longer confined to a mere acceleration of the breathing. This symptom still continues in an aggravated degree, but the noise accompanying the respiration has gradually assumed a very different character from that which marked it at first: each respiration is now attended by a loud crouping noise, which might be heard in an adjoining apartment. The child has frequent attacks of convulsions, during which the features are much distorted. These convulsive paroxysms vary in violence and in duration in different cases; sometimes the whole body is affected. In the child of a Mons. Lambert, in whom the convulsions were frequent and severe, the state of opisthotonos was so complete, that for many days the head and heels were the only parts which touched the bed: if, with difficulty, this apparently painful position was altered by the mother, it was quickly resumed. The brow of the child is generally knit. The anxiety of the countenance is extreme. There is no febrile action in the system to be detected. No determination of blood to the head is manifested, either by an increase of heat or a flushed countenance.

I have known the firm contraction of the thumb, the rigidly bent position of the hand and foot, and the crouping noise in respiration, continue for many weeks without intermission. The child sometimes appears lively: its countenance will be animated by a momentary cheerfulness; but it almost invariably awakens from its slumbers, however tranquil they may be apparently, with a convulsive paroxysm, similar to that which I have described. The paroxysm having terminated, the child appears much exhausted, and almost motionless for some time. Dr. Clarke observes, that the term of chronic croup has been sometimes applied to this affection; "but it is very different from croup, and is altogther of a convulsive character."

In many instances, it may be difficult, or perhaps impossible, to assign the probable cause of the affection I have described. I believe it not unfrequently arises from painful dentition. I

have known all the above symptoms gradually disappear upon the appearance of teeth. Such was the case in the child of a Mr. Prall. It is worthy of observation, also, that another child of Mr. Prall's, otherwise in apparent good health, who has not yet cut a tooth, inspires with the much-talked-of "crouping noise," which, with many practitioners, is alone considered a sufficient signal for immediate bleeding, &c. &c. &c.

The treatment demanded in the above state may be very briefly laid down. The gums should be freely lanced, if they appear swoln or inflamed. To keep the bowels open, purgatives must be freely given: calomel, in combination with powder of jalap, is perhaps the best remedy. During the convulsive paroxysm, the child should be put into a warm bath. The diet, if the child unfortunately is deprived of its mother's breast, should be very strictly attended to. The digestive powers are evidently weakened, and it is destructive to impose upon the stomach a task which it is not capable of performing, by giving solid food in any form.

I have known no instance in which this convulsive affection has terminated fatally, or in which it has induced any more serious disease. It is true the convulsive paroxysms have returned at intervals for a considerable period. If, however, I am correct in considering dentition as a frequent cause, the long continuance of the symptoms is readily explained; they cannot be expected to cease so long as the exciting cause continues. In some cases, a free action of the bowels puts a stop to all the symptoms for a time. I have rarely, if ever, known them subside entirely, until the process of dentition has been sufficiently advanced to warrant the free division of the gums, or till teeth have passed through by the efforts of nature.

I do not mean to assert that the symptoms vanish instantaneously, as if by magic, upon the appearance of a single tooth;—they pass off gradually. When convulsive affections have once been induced, they remain for some time as the effect of habit, even if the original cause be entirely removed.

I am not prepared to contend, that the convulsive affection which I have described is *totally* independent of the brain; but I do maintain, that we are not justified in inflicting upon a child, labouring under the above symptoms, even collectively, and much less individually, an active and severe mode of treatment. We have no right to presume that such a state must necessarily be followed by any alarming affection of the head. I do not deny that the brain *may* become the seat of disease during the progress of this affection. He who is at all conversant with the diseases of children, will always bear in mind the disposition that exists to affections of the head. He will be prepared, it is true, to attack the disease before it shall have advanced so far as to defy his best-directed efforts; but he will not rashly seize upon an individual symptom, even if it should be a "crouping noise," "a bent thumb," or "a dilated pupil," and create unnecessary apprehension, by prognosticating the occurrence of that frequent bugbear—hydrocephalus, unless instant recourse be had to bleeding to faintness, blisters, starvation, and three or four grains of calomel every three hours. I must be allowed to observe, that I protest against no imaginary error. It is the favourite doctrine of those whose opinions are justly considered worthy of attention, that the occurrence of any one of the above symptoms "is always followed by water in the brain;" and hence, I think, arises the ready explanation of the wonderful success of some individuals in the treatment of hydrocephalus. A particular symptom makes its appearance,—hydrocephalus is said to be at hand: instant recourse is had to a formidable, and, I believe, unnecessary mode of treatment. The child recovers, notwithstanding even the severity of the practice, and the case is registered as a creditable proof of the cure of this formidable disease.

*Seymour-place, Bryanstone square.*

